# Preparation of Chitosan/Clay Composites for Safe and Effective Hemorrhage Control

**DOI:** 10.3390/molecules27082571

**Published:** 2022-04-15

**Authors:** Zhiyuan Yang, Tong Ye, Fei Ma, Xinhong Zhao, Lei Yang, Guifang Dou, Hui Gan, Zhuona Wu, Xiaoxia Zhu, Ruolan Gu, Zhiyun Meng

**Affiliations:** 1 College of pharmacy, Henan University, Kaifeng 475000, China; 104754191128@henu.edu.cn (Z.Y.); douguifang@vip.163.com (G.D.); 2 Beijing Institute of Radiation Medicine, Beijing 100850, China; 17864291181@163.com (T.Y.); jinxingma@126.com (F.M.); 18754807130@163.com (X.Z.); ziwangfengling@sina.com (L.Y.); ganh2003@163.com (H.G.); wznphd@126.com (Z.W.); 13681022512@163.com (X.Z.)

**Keywords:** chitosan, kaolin, montmorillonite, hemostatic material, hemorrhage control

## Abstract

Uncontrolled hemorrhage from trauma or surgery can lead to death. In this study, chitosan/kaolin (CSK) and chitosan/montmorillonite (CSMMT) composites were prepared from chitosan (CS), kaolin (K), and montmorillonite (MMT) as raw materials to control bleeding. The physiochemical properties and surface morphology of CSK and CSMMT composites were analyzed by Fourier transform infrared spectrometry (FT-IR), X-ray diffraction (XRD), scanning electron microscopy (SEM), zeta potentials, and X-ray fluorescence (XRF). The hemostatic mechanism was measured in vitro by activated partial thromboplastin time (APTT), prothrombin time (PT), in vitro clotting time, erythrocyte aggregation, and thromboelastogram (TEG). The hemostasis ability was further verified by using tail amputation and arteriovenous injury models in rats. The biocompatibility of CSK and CSMMT was evaluated by in vitro hemolysis, cytotoxicity assays, as well as acute toxicity test and skin irritation tests. The results show that CSK and CSMMT are promising composite materials with excellent biocompatibility and hemostatic properties that can effectively control bleeding.

## 1. Introduction

Hemorrhage is the leading cause of death from natural disasters, traffic accidents, and other civilian trauma, and is the most common cause of death during orthopedic, cardiovascular, and liver operations [1,2,3]. Therefore, hemostasis is a critical step in emergency medical treatment. Currently, hemostatic materials on the market mainly include zeolite powder, starch hemostatic microspheres, medical gelatin sponges, chitosan bandages, fibrin dressing, oxidized cellulose, oxidized regenerated cellulose dressings, etc. [4,5]. Although these materials are effective in bleeding control, each of them possesses shortcomings. For example, fibrin glues derived from blood components are expensive and susceptible to infection [6,7]; collagen can activate platelets to stop hemorrhage, but its hemostasis effect is limited by its poor tissue adhesion [8,9]; and porous zeolite releases heat during the application, resulting in skin and tissue damage [10,11]. Therefore, efficient and safe hemostatic agents are needed. An ideal hemostasis agent is required that can control blood loss quickly and effectively, with the characteristics of high safety, convenient use, and a low price [12].

Chitosan, a natural polycationic polysaccharide commercially obtained from the deacetylation of chitin [13], functions as a hemostatic and bacteriostatic agent, leading to reduced blood loss and promoted tissue healing [14,15]. At the same time, it is a multifunctional material with excellent biocompatibility and is nonimmunogenic and nonirritating [16]. Chitosan with positively charged free amino ions can induce red blood cell (RBC) aggregation around wound sites through electrostatic interaction with negatively charged neuraminic acid residues on the surface of the RBC membrane, thus causing blood coagulation [17]. In recent years, hemostatic agents using chitosan and its derivatives as the main hemostatic materials have been widely applied in the field of hemostasis. There are various Food and Drug Administration-approved chitosan hemostasis dressings, such as Celox hemostasis powder and HemCon chitosan hemostasis bandages [18,19,20,21]. However, how to improve its hemostatic potential remains a challenge.

Clay is a layered silicate composed of silicon-oxygen tetrahedron and aluminum-oxygen octahedron. According to the ratio of tetrahedral lamella to octahedral lamella, clays are mainly divided into two types: 1:1 clay (kaolin) [22], and 2:1 clay (montmorillonite) [23]. Kaolin and montmorillonite can induce the human blood coagulation cascade reaction by rapidly absorbing the water of the wound blood, enriching platelets and clotting factors, and forming a clay layer that seals the wound to stop the bleeding. Kaolin activates blood coagulation factor XII [24,25], and montmorillonite activates blood coagulation factors VII, VIII, and XI [26]. However, the hemostasis of clay depends on the coagulation system, and it cannot control massive bleeding in coagulopathy. In addition, there are certain safety problems with clay hemostatic agents, such as the hemolysis of kaolin and montmorillonite [27].

In this study, CSK and CSMMT hemostatic materials were prepared by combining chitosan with kaolin and montmorillonite, respectively. In the past, the research on chitosan mainly focused on the chemical modification of chitosan structure or on composites of chitosan with other organic materials [28,29]. In recent years, there have been some literature reports on the preparation of gauze or microspheres by the blending of chitosan and kaolin. These physical mixed materials may have problems of inhomogeneity, and the toxicity of clay has not been eliminated [30,31]. Differently from trivial physical mixtures, the CSK and CSMMT prepared by us were combined through intermolecular force (hydrogen bond), which can enhance the hemostatic effect and overcome the safety problem of clay through the synergistic effect of chitosan absorbing red blood cells and clay activating coagulation factors. As a composite multifunctional hemostatic material, the physiochemical properties of the composite material were characterized, including FT-IR, XRD, SEM, XRF, and Zeta potential. APTT, PT, erythrocyte aggregation, and TEG were used to study the hemostatic mechanism of composite materials, and further animal experiments were conducted to verify the hemostatic effect of composite materials. Meanwhile, a comprehensive safety evaluation of the composite was conducted, including hemolysis, in vitro cytotoxicity, acute toxicity and skin irritation tests.

## 2. Results and Discussion

### 2.1. Characterization

#### 2.1.1. FTIR and XRD Analyses

**Figure 1:**
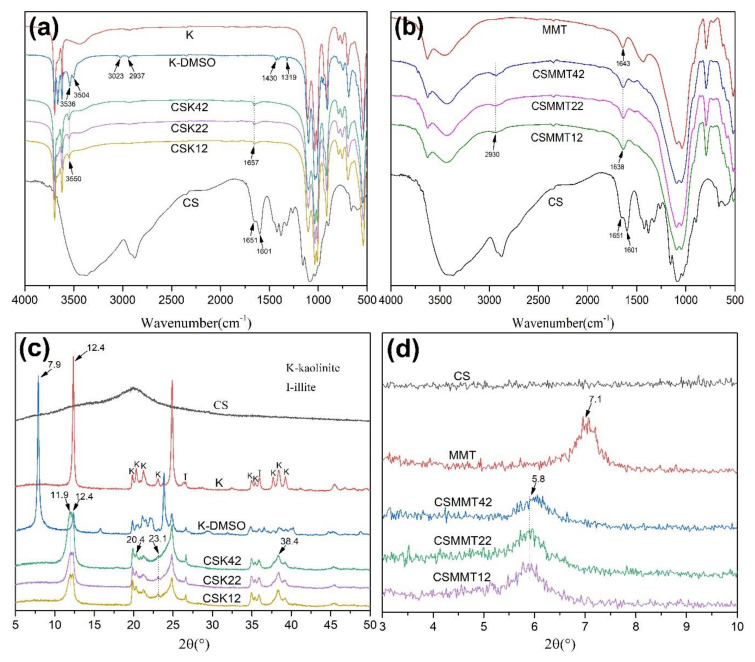
FTIR spectra (**a**,**b**) and XRD patterns (**c**,**d**) of CS, K, K−DMSO, CSK42, CSK22, and CSK12 (**a**,**c**); and MMT, CSMMT42, CSMMT22, and CSMMT12 (**b**,**d**).

The FTIR spectra of CS, K, K−DMSO, CSK42, CSK22, and CSK12 are shown in Figure 1a. The spectrum of CS powder exhibits bands at 1651 and 1601 cm^−1^, respectively, attributed to C=O stretching vibration (amide I band) and N-H stretching vibration (amino II band). The vibration bands of K−DMSO at 3536 and 3504 cm^−1^ were attributed to the formation of hydrogen bonds between the S=O bond in DMSO and -OH between the kaolin layers. The vibration bands at 3023 and 2937 cm^−1^ were attributed to the stretching vibration of C-H. Bands at 1430 and 1319 cm^−1^ were attributed to the bending vibration of C-H [32]. The vibration band of CSK at 3550 cm^−1^ was attributed to the formation of the hydrogen bond between -NH_2_ of chitosan and -OH of kaolin, and the C=O stretching vibration band of CS at 1651 cm^−1^ was shifted to 1657 cm^−1^. This indicates that chitosan reacts with kaolin successfully. The FTIR spectra of CS, MMT, CSMMT42, CSMMT22, and CSMMT12 are shown in Figure 1b. For CSMMT, the broad vibration bands at 2930 cm^−1^ were attributed to the stretching vibration band of C-H. The vibration band at 1638 cm^−1^ was speculated to overlap with the -OH bending vibration of montmorillonite and the chitosan amino band, indicating a negative ion interaction between the chitosan amino and the montmorillonite layer [33,34].

To confirm whether CSK and CSMMT are intercalated structures, the XRD patterns of CS, K, K−DMSO, CSK42, CSK22, and CSK12 are shown in Figure 1c and CS, MMT, CSMMT42, CSMMT22, and CSMMT12 are shown in Figure 1d. According to Bragg’s Law (2dsinθ = nλ), the interval between layers can be calculated [32,33,34]. The expansion of the K−DMSO interlayer space was attributed to the intercalation effect of DMSO, which shifts from 2θ = 12.4 (001) to 7.9, and the interlayer space expanded from 0.72 to 1.12 nm, indicating that DMSO intercalation is successful [32]. Basically, the 2θ = 12.4 (001) of CSK was the same before and after the reactions. However, CSK showed changes in the characteristic peaks of kaolin at 2θ = 11.9, 20.4, 23.1, and 38.4. Combined with the FTIR results, it was attributed to the formation of hydrogen bonds between chitosan and the surface hydroxyl groups of kaolin. In contrast, the 2θ = 7.1 (001) of CSMMT was shifted to 5.8, and the interlayer spacing expanded from 1.24 to 1.51 nm, indicating the success of chitosan intercalation [33,34].

#### 2.1.2. SEM Observation

**Figure 2:**
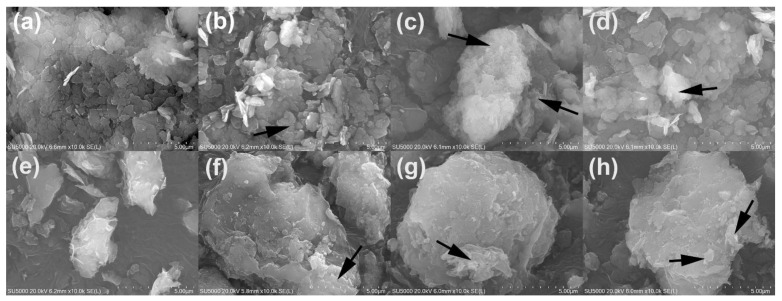
SEM images of (**a**) K, (**b**) CSK42, (**c**) CSK22, (**d**) CSK12, (**e**) MMT, (**f**) CSMMT42, (**g**) CSMMT22, and (**h**) CSMMT12.

SEM was applied to observe the morphological changes of the material before and after the modification. The SEM of K, CSK42, CSK22, and CSK12 are shown in Figure 2a–d. Kaolin is mainly a lamellar stacking structure, with a particle size of less than 1 μm and a smooth surface [32]. The lamellar structures of CSK42, CSK22, and CSK12 were not damaged after the modification. The surfaces of the composites were covered by small particles, indicating that chitosan covered the surface of kaolin. The SEM of MMT, CSMMT42, CSMMT22, and CSMMT12 are shown in Figure 2e–h. The lamellar structure of montmorillonite is weaker than that of kaolin, and it is mainly a massive structure with a particle size of less than 5 μm and a smooth surface [34]. The modified CSMMT42, CSMMT22, and CSMMT12 were still massive structures with a particle size of about 5 μm. There were few particles on the surface, indicating that a small amount of chitosan from the reaction system adhered to the surface of montmorillonite.

#### 2.1.3. Zeta Potential Analysis

**Figure 3:**
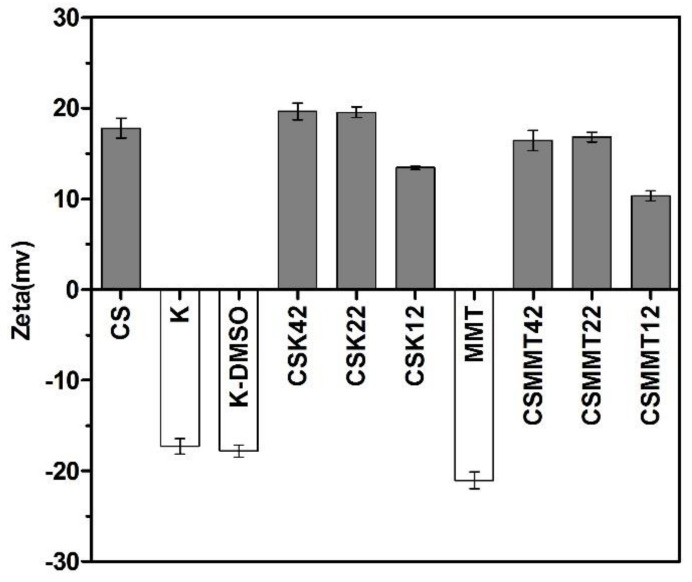
Zeta potentials of CS, K, K−DMSO, CSK42, CSK22, CSK12, MMT, CSMMT42, CSMMT22, and CSMMT12. Data represent the mean ± SD (*n* = 3).

The zeta potential can reflect the charge of dispersed particles. There are many free positively charged amino groups on the chitosan surface, and the positive charge of the amino group can adsorb RBCs and platelets in the blood through electrostatic interaction [17]; this is also the mechanism for the hemostasis of chitosan. The silicon-oxygen tetrahedron Si^4+^ in kaolin and montmorillonite was replaced by Al^3+^, and the aluminum-oxygen octahedron Al^3+^ was replaced by Mg^2+^ and Fe^2+^, making the crystal layer generate excess negative charge. This phenomenon is called permanent negative charge [35,36]. The zeta potential of the test composites is shown in Figure 3. The charge values of CSK42 and CSK22 were +19.5 mv and +19.3 mv, respectively, whereas the charge value of CSK12 was +13.4 mv, lower than CSK42 and CSK22. The reason for this phenomenon may be that the ratio of chitosan to kaolin was already saturated (1:1, *w*/*w*), and the excessive amount of chitosan had no obvious effect on the charge of CSK. The charge values of CSMMT42 and CSMMT22 were +16.4 mv and +16.8 mv, respectively, whereas the charge value of CSMMT12 was +10.3 mv, obviously different from CSMMT42 and CSMMT22. The reason was the same as for CSK. In general, the products all showed positive charges after the reaction of chitosan with kaolin and montmorillonite. With the increase of positive charge, the adsorption capacity of RBCs and platelets can be enhanced, thus improving the hemostatic ability of the samples.

#### 2.1.4. Chemical Composition Analysis

The XRF analysis results are shown in Table 1. The sulfur content in K−DMSO increased to 3.95, indicating that DMSO intercalation was successful, consistently with the FTIR and XRD results. The sulfur in CSK42, CSK22, and CSK12 decreased to about 0.11, indicating that about 98% of K−DMSO was de-embedded and reacted with chitosan. There are exchangeable Ca^2+^ and Na^+^ between montmorillonite layers [37], Ca^2+^ decreased from 1.38 to 0.51, and Na^+^ decreased from 1.59 to 0.46 in CSMMT42, CSMMT22, and CSMMT12, indicating that chitosan replaces Ca^2+^ and Na^+^ between montmorillonite layers.

### 2.2. Hemostatic Mechanisms

#### 2.2.1. APTT and PT Measurements

**Figure 4:**
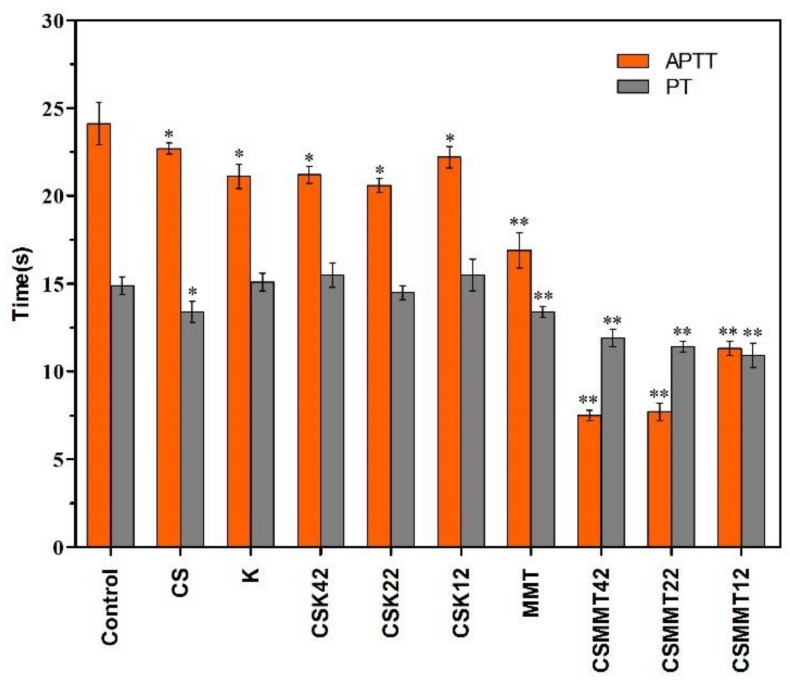
APTT and PT of control, CS, K, CSK42, CSK22, CSK12, MMT, CSMMT42, CSMMT22, and CSMMT12. Data represent the mean ± SD (*n* = 5). * *p* < 0.05; ** *p* < 0.01, compared to control.

In the clinic, APTT and PT are usually used as two key indexes to evaluate the intrinsic and extrinsic coagulation pathways, respectively [38]. As shown in Figure 4, compared to blank plasma, other materials shortened the APTT value statistically. Montmorillonite and CSMMT shortened the PT value, whereas kaolin and CSK did not. The results show that kaolin mainly only acted on endogenous coagulation pathways, consistent with the literature reporting that kaolin mainly activates factor XII [24,25]. However, montmorillonite could activate factors VII, VIII, and XI [26]. Factors VII and VIII can act on exogenous pathways, while XI acts on endogenous pathways.

#### 2.2.2. Whole Blood Clotting

**Figure 5:**
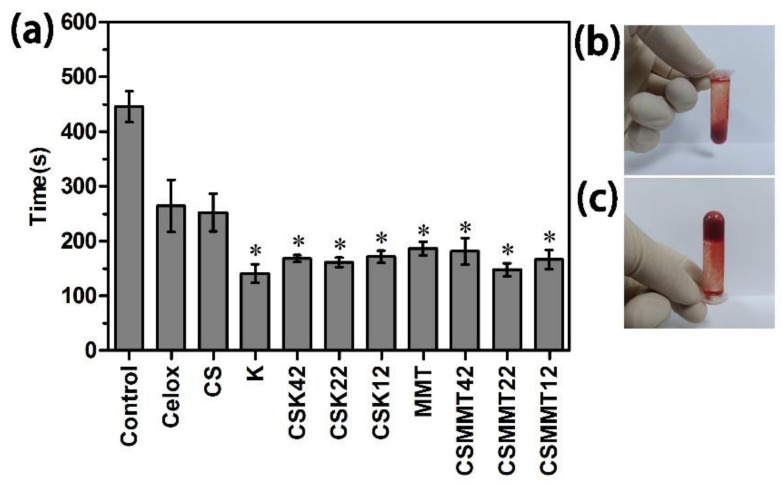
(**a**) In vitro blood clotting time of control, Celox, CS, K, CSK42, CSK22, CSK12, MMT, CSMMT42, CSMMT22, and CSMMT12. Data represent the mean ± SD (*n* = 3). * *p* < 0.05 compared to Celox. (**b**,**c**) Coagulation state of in vitro sample.

In vitro blood clotting time is mainly used to evaluate the hemostatic ability of materials. The results for in vitro blood clotting time are shown in Figure 5. As per the standard of complete blood coagulation (Figure 5b,c), the normal clotting time of blank rabbit blood was about 450 s. Compared to the blank group, the clotting time of rabbit blood treated with all test materials (Celox, K, CSK42, CSK22, CSK12, MMT, CSMMT42, CSMMT22, and CSMMT12) was significantly reduced. Compared to the positive control (Celox), the clotting time of rabbit blood treated with test materials was significantly shortened. The shortened clotting time was mainly because CSK and CSMMT can absorb water, while the positive charge of CSK and CSMMT combined with negatively charged RBCs, thus promoting blood coagulation. The results show that CSK22 and CSMMT22 are the best among the test materials. Based on the comprehensive results of APTT, PT, in vitro coagulation time, and physiochemical properties, the ratio of chitosan to kaolin was already saturated (1:1, *w*/*w*), and excessive chitosan has no obvious promoting effect on hemostasis. Therefore, CSK22 and CSMMT22 were selected for the next safety and in vivo hemostasis studies.

#### 2.2.3. Erythrocyte Aggregation Test

**Figure 6:**
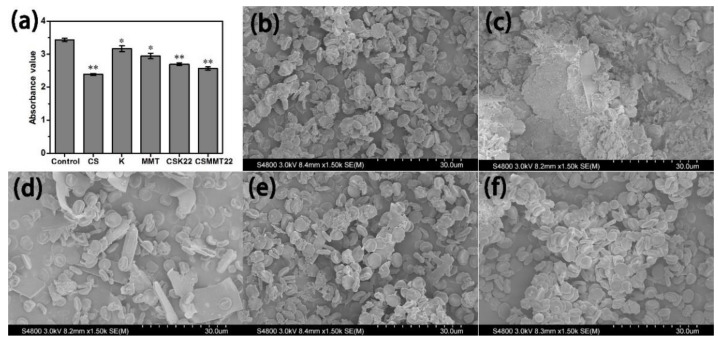
(**a**) RBC adhesion of Control, CS, K, MMT, CSK22, and CSMMT22. Data represent the mean ± SD (*n* = 3). * *p* < 0.05; ** *p* < 0.01 compared to Control. SEM images of (**b**) CS, (**c**) K, (**d**) MMT, (**e**) CSK22, (**f**) CSMMT22.

The aggregation results of erythrocytes are shown in Figure 6a. Chitosan has free amino groups that can quickly adsorb RBCs. Compared with kaolin and montmorillonite, the adsorption capacity of CSK22 and CSMMT22 has enhanced effects, which is consistent with the positive charge of CSK22 and CSMMT22 in Zeta potential. The enhancement of the RBC adsorption capacity of CSK22 and CSMMT22 can be seen more intuitively by SEM. As shown in Figure 6b–f, a small number of RBCs on the surface of montmorillonite were deformed and broken, suggesting that montmorillonite might cause hemolysis. The RBCs on the surface of CSK22 and CSMMT22 were significantly more numerous than for kaolin and montmorillonite. Erythrocyte aggregation test results showed that CSK22 and CSMMT22 could improve the adsorption capacity of RBCs through the effect of electric charge.

#### 2.2.4. Thromboelastograph Analysis

TEG analysis mainly reflects the dynamic changes of blood coagulation and can comprehensively monitor the whole process of coagulation factors, platelets, and fibrinogen. The R value indicates the initial time of clot formation, while α is related to clotting rate and represents the rate at which fibrin forms and binds into a network. The MA value represents the maximum strength of the clot; platelet contribution is about 80%, and fibrin contribution is about 20%, so the MA value is the best reflection of the platelet aggregation function. As shown in Table 2, compared with the negative control, the R values of CSK22 and CSMMT22 decreased, while the α Angle and MA values significantly increased, indicating that CSK22 and CSMMT22 can activate coagulation factors and platelets, accelerate the formation and networking of fibrin, and then appear in a hypercoagulant state [39].

### 2.3. Hemostasis In Vivo

#### 2.3.1. Hemostatic Efficiency on Rat Tail Amputation

**Figure 7:**
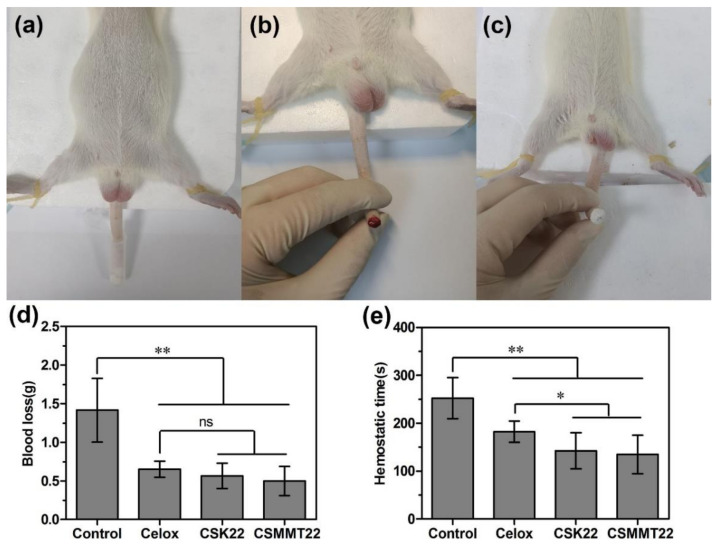
(**a**) Photographs of the rat tail amputation model, (**b**) amputation of tail to cause hemorrhage, (**c**) hemostatic effect by contacting the wound, (**d**) blood loss and (**e**) hemostatic time of control, Celox, CSK22, and CSMMT22. Data represent the mean ± SD (*n* = 6). * *p* < 0.05; ** *p* < 0.01; ns *p* > 0.05, compared with the two groups.

The hemostatic performance of CSK22 and CSMMT22 was evaluated preliminarily with the rat tail amputation model. Blood loss and hemostasis time were used as the main evaluation indexes. Blank gauze was used as the negative control group, and Celox powder was used as the positive control group. As shown in Figure 7, blood loss with Celox (0.65 g), CSK22 (0.57 g), and CSMMT22 (0.50 g) was significantly reduced compared to the control group (1.42 g). There were no significant differences in blood loss between CSK22 and CSMMT22 compared to Celox. Moreover, the hemostasis times of Celox (182 s), CSK22 (142 s) and CSMMT22 (135 s) were significantly shorter than that of Control (253 s). The hemostasis time of CSK22 and CSMMT22 was statistically shorter than that of Celox. The results of the rat tail amputation model indicate that CSK22 and CSMMT22 showed excellent hemostasis.

#### 2.3.2. Hemostatic Efficiency on Rat Arteriovenous Hemorrhage

**Figure 8:**
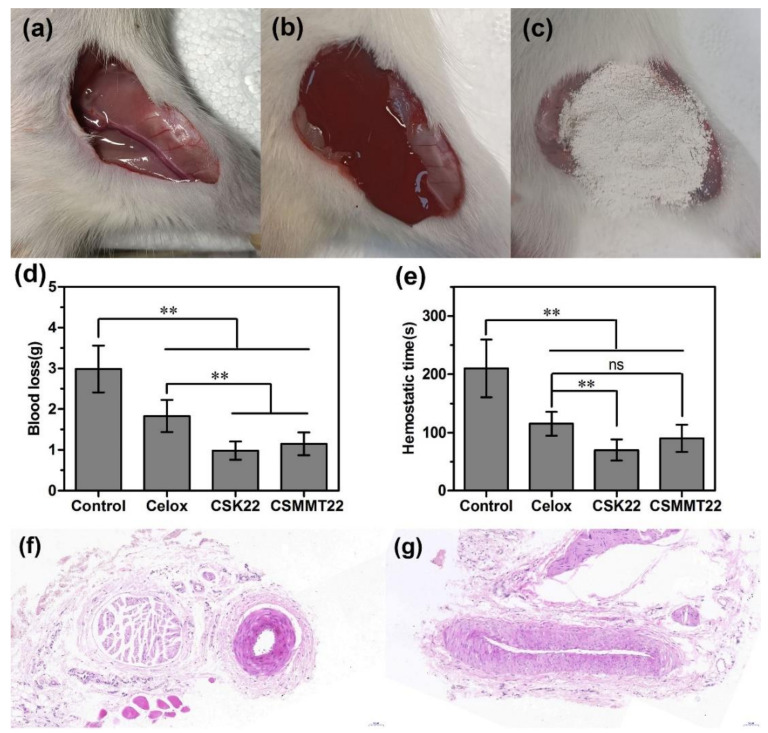
(**a**) Photographs of the rat femoral arteriovenous injury model, (**b**) transected arteriovenous that caused hemorrhage, and (**c**) staunch bleeding after filling the wound; (**d**) blood loss and (**e**) hemostatic time of control, Celox, CSK22, and CSMMT22. Data represent the mean ± SD (*n* = 6). ** *p* < 0.01; ns *p* > 0.05, compared with the two groups. Histological analysis of rat femoral arteriovenous injury (magnification, ×20), (**f**) arteriovenous treated with CSK22, and (**g**) arteriovenous treated with CSMMT22.

The hemostatic performance of CSK22 and CSMMT22 was further tested and evaluated with the rat femoral arteriovenous injury model. Blank gauze and Celox powder were used as the negative and positive controls, respectively. Blood loss and hemostasis time were used as evaluation indexes. As shown in Figure 8, blood loss with Celox (1.8 g), CSK22 (1.0 g) and CSMMT22 (1.2 g) was significantly reduced compared to Control (3.0 g). Blood loss with CSK22 and CSMMT22 was also significantly reduced compared to Celox. Moreover, the hemostasis time of Celox (115 s), CSK22 (70 s) and CSMMT22 (90 s) was significantly shorter than that of the control (210 s). The hemostasis times of CSK22 and CSMMT22 were shorter than that of Celox, but the hemostasis time of CSMMT22 showed no significant difference compared to Celox. Lastly, a histological analysis of femoral arteriovenous vessels in rats was carried out. After fixing in paraffin, femoral vessels were stained with HE for histological analysis, as shown in Figure 8f,g. Histological analysis indicated that there was no inflammatory infiltration or pathological degeneration in all test groups. The above hemostatic performance evaluation indicated that CSK22 and CSMMT22 had better hemostatic performance than Celox, which may be due to the synergistic effect of the composite materials on the enrichment of RBCs and platelets, and the activation of coagulation factors.

### 2.4. Biocompatibility Evaluation

#### 2.4.1. Hemolytic Tests In Vitro

**Figure 9:**
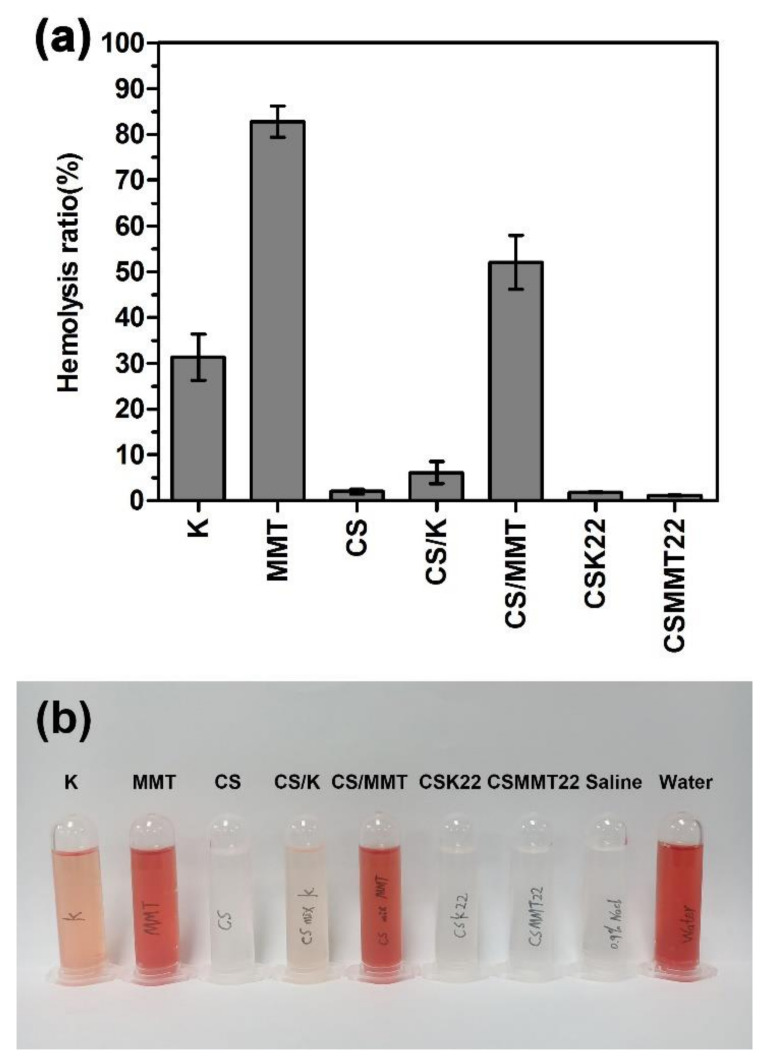
(**a**) Hemolysis rates of K, MMT, CS, CS/K, CS/MMT, CSK22, and CSMMT22. Data represent the mean ± SD (*n* = 3). (**b**) Photograph of RBCs treated with K, MMT, CS, CS/K, CS/MMT, CSK22, CSMMT22, saline, and water, respectively.

The blood compatibility of K, MMT, CS, CS/K, CS/MMT, CSK22, and CSMMT22 was evaluated by in vitro hemolysis, as shown in Figure 9. The hemolytic rates of kaolin, montmorillonite, CS/K, and CS/MMT were 31%, 82%, 7%, and 52%, respectively. In contrast, the hemolysis rates of CS, CSK22 and CSMMT22 were all less than 5%, much lower than kaolin and montmorillonite, suggesting that the formation of composite materials reduced the destruction of RBCs [40]. The reduced hemolysis rate of CSK22 was due to the physical barrier formed by the interaction of chitosan with kaolin, which reduces the direct contact between kaolin and RBCs. The decreased hemolysis rate of CSMMT22 may be due to the change in the properties of montmorillonite caused by the intercalation of chitosan with montmorillonite. On the other hand, the adhesion of a small amount of chitosan on the surface of montmorillonite also has a certain protective role.

#### 2.4.2. Cytotoxicity Assay

**Figure 10:**
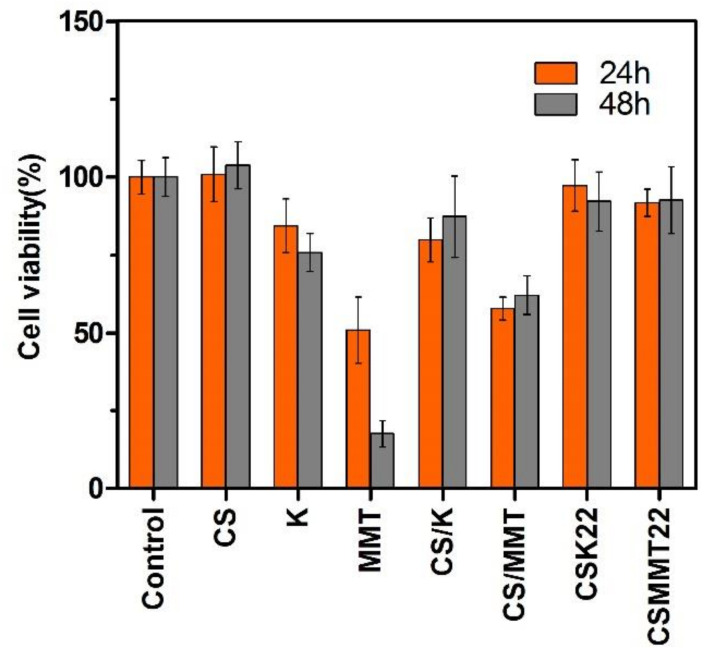
Cytotoxic effects of the control, CS, K, MMT, CS/K, CS/MMT, CSK22, and CSMMT22. Data represent the mean ± SD (*n* = 6).

In vitro cytotoxicity analysis is widely used to determine the influence of biological materials on cell growth. In general, biomaterials are considered safe with cell activity values of more than 75% [41,42]. In Figure 10, the cell activity values of MMT and CS/MMT are show to be 50.9% and 57.8% at 24 h, and 17.6% and 62.1% at 48 h, respectively, suggesting that MMT was cytotoxic to L929 cells. However, the cell activity values of CS, K, CSK22, and CSMMT22 are cytocompatible (>75%). The result suggests that the addition of chitosan facilitated the formation of a sandwiched structure that can reduce the cytotoxicity of MMT.

#### 2.4.3. Acute Toxicity Test

**Figure 11:**
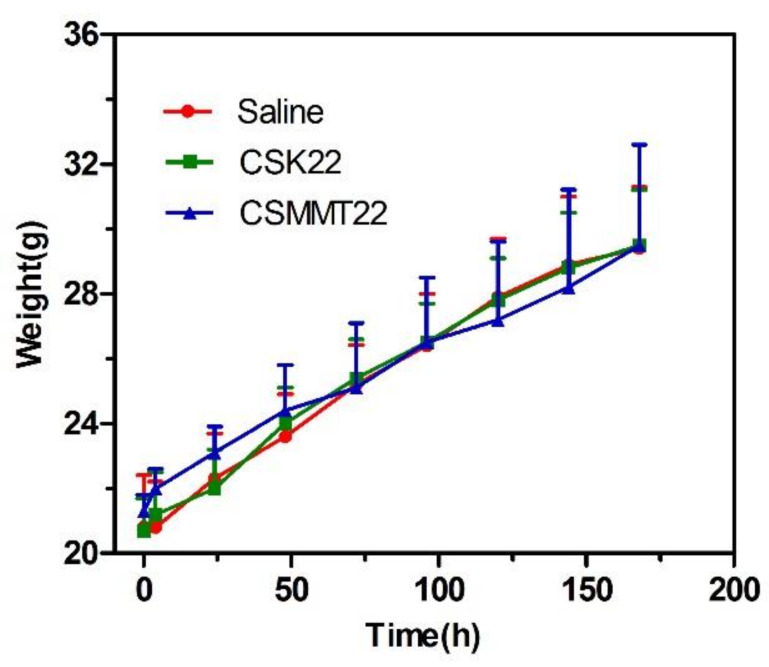
Acute toxicity test results of saline, CSK22, and CSMMT22. Data represent the mean ± SD (*n* = 6).

After intraperitoneal injection of saline and experimental sample extract, kunming mice were generally in good condition for 7 consecutive days, without death, convulsion or prone position. As shown in Figure 11, the changes of body weight in the CSK22 and CSMMT22 groups were consistent with that in the saline group, showing a steady increase. The results show that CSK22 and CSMMT22 did not cause acute toxicity.

#### 2.4.4. Skin Irritation Test

**Figure 12:**
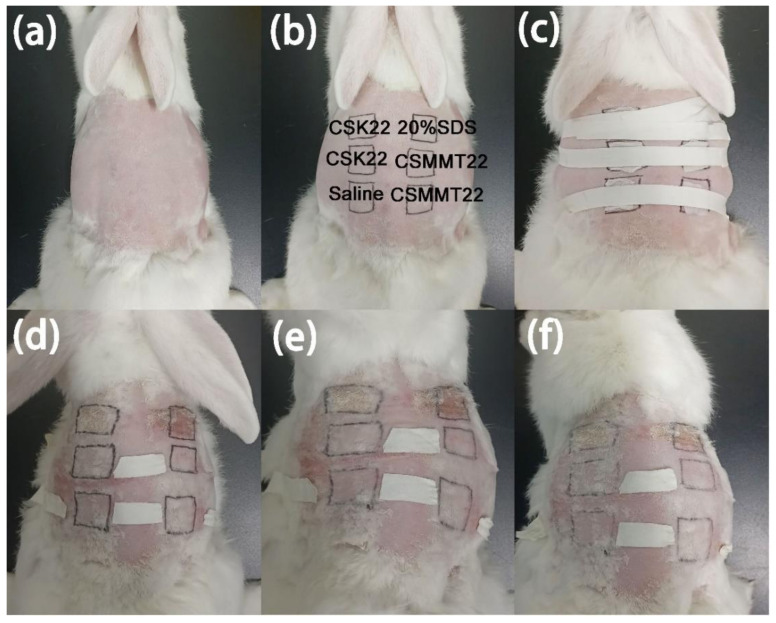
(**a**) Exposed skin, (**b**) experimental area for each sample, (**c**) medical tape fixation, (**d**) 24 h after patch removal, (**e**) 48 h after patch removal, (**f**) 72 h after patch removal.

Skin stimulation tests are mainly performed to test whether hemostatic materials will produce erythema and edema when they are in contact with the skin, causing a stimulating reaction. As shown in Figure 12, there was obvious erythema and edema in the 20% SDS test area, while the CSK22 and CSMMT22 experimental groups showed no obvious erythema and edema, as did the saline group. The experimental results show that CSK22 and CSMMT22 hemostatic materials produced no irritation on the skin of New Zealand rabbits.

The results show that kaolin and montmorillonite showed significant hemolysis and cytotoxicity, while CSK22 and CSMMT22 had good biocompatibility. It has been reported in the literature that interfacial interactions can improve the toxicity of inorganic materials [43]. We speculated that chitosan in CSK22 and CSMMT22 eliminated the hemolysis of clay through an interfacial interaction. Meanwhile, the literature shows that chitosan can promote cell proliferation [44,45,46].We speculated that the high cell viability in the CSK22 and CSMMT22 treatment groups could be attributed to the proliferation promotion effect of chitosan in CSK22 and CSMMT22. These results indicate that CSK22 and CSMMT22 have no hemolysis and cytotoxicity, and can be used as potential hemostatic agents.

## 3. Materials and Methods

### 3.1. Materials

Chitosan powder (degree of deacetylation ≥ 95%, low viscosity, 100–200 mPa·s) and kaolin particles were purchased from Shanghai Aladdin Co., Ltd. Sodium montmorillonite (Na-MMT, specific surface area 240 m^2^/g) was purchased from Shanghai Yuanye Bio-Technology Co., Ltd. (Shanghai, China). Acetic acid, DMSO, isopropanol, and phenol were all purchased from Sinopharm Chemical Reagents Co., Ltd (Beijing, China). Dulbecco’s Modified Eagle’s Medium-high glucose (DMEM), Dulbecco’s phosphate-buffered saline (DPBS), and penicillin-streptomycin were purchased from Sigma-Aldrich (Shanghai, China). Fetal bovine serum (FBS) and 0.25% trypsin-EDTA were purchased from Thermo Fisher Scientific (Shanghai, China). Furthermore, 3-(4,5-dimethyl-2-thiazolyl)-2,5-diphenyl-tetrazolium bromide (MTT) was purchased from Beijing Solarbio Science Co., Ltd. (Beijing, China). A mouse fibroblast cell line (L929) was obtained from the American Strain Preservation Center. Healthy male New Zealand rabbits with weights of ca. 2.0~2.5 kg and healthy Sprague-Dawley male rats with weights of ca.180~220 g were purchased from Beijing Keyu Experimental Animal Co., Ltd. (Beijing, China). Two rats per cage and one New Zealand rabbit per cage were raised at temperature of 25 °C and a humidity of 50%RH. All animal procedures were performed in accordance with the ARRIVE guidelines and approved and reviewed by the Beijing Institute of Radiation Medicine (Beijing, China, IACUC-DWZX-2020-503).

### 3.2. Preparation of CSK Composites

Kaolin was vacuum-dried at 100 °C for 6 h. Kaolin, DMSO, and deionized water were mixed at a ratio of 1:12:1.2 (*w*/*v*/*v*) and placed in a constant temperature magnetic stirrer for 24 h at 60 °C. After the reaction, the product was washed with hot anhydrous ethanol thrice to remove the residual DMSO in the product. The product was vacuum-dried at 60 °C for 12 h, and coded as K−DMSO.

Chitosan was added to 1% (*v*/*v*) acetic acid solution to prepare 0.5%, 1%, and 2% (*w*/*v*) solutions, respectively. K−DMSO was added to chitosan solutions of different concentrations and placed in a constant temperature magnetic stirrer for reaction at 80 °C for 12 h. After the reaction, the product was centrifuged, washed with distilled water thrice, and filtered. The product was vacuum-dried at 60 °C for 12 h. The composites with CSK ratios of 1/2, 2/2, and 4/2 were coded as CSK12, CSK22, and CSK42, respectively.

### 3.3. Preparation of CSMMT Composites

The sodium montmorillonite (Na-MMT) was dispersed in deionized water to prepare a 2% (*w*/*v*) suspension liquid. The suspension liquid was placed in a flask and swelled for 2 h under a magnetic stirrer at 60 °C. Chitosan was added to 1% (*v*/*v*) acetic acid solution to prepare 0.5%, 1%, and 2% (*w*/*v*) solutions, respectively. The solutions were slowly added to the montmorillonite suspension liquid, and magnetically stirred at 60 °C for 6 h. After the reaction, the product was centrifuged, washed with distilled water thrice, and filtered. The product was vacuum-dried at 60 °C for 12 h. The composites with CSMMT ratios of 1/2, 2/2, and 4/2 were coded as CSMMT12, CSMMT22, and CSMMT42, respectively.

### 3.4. Characterization of CSK and CSMMT Composites

A Nicolet 6700 Fourier transform infrared spectrometer (FTIR Nicolet IS5, Thermo Fisher Scientific, Waltham, MA, USA) was used to measure the infrared spectra of the composites. The spectra were recorded in a wavenumber range from 500 to 4000 cm^−1,^ with 32 scans per spectrum and a resolution of 4 cm^−1^. X-ray diffraction of the samples was performed on a diffractometer (XRD smartlab9k, Rigaku, Japan), which was operated at 40 kV and 40 mA with a Cu Kα (λ = 1.54 Å) radiation. The samples were air-desiccated and placed on the XRD spinner, followed by scanning for 2θ ranging from 3°to 50°.

Because the material itself does not have electrical conductivity, the samples were examined by field emission scanning electron microscopy (SEM Ultim Max, Oxford Instruments, UK) after gold coating. The zeta potentials of the samples were determined using a Zetasizer (Nano S90, Malvern, UK). The samples were dispersed in deionized water and sonicated for 5 min before the measurements. The chemical compositions of the samples were measured with an X-ray fluorescence (XRF) spectrometer (XRF ARLAdvant’X Intellipower 3600, Thermo Elemental, Waltham, MA, USA) operated at 50 kV and 50 mA with a Lawrencium target.

### 3.5. In Vitro Blood Plasma Coagulation Assay

Fresh whole blood of Sprague-Dawley rats was collected and anticoagulated with 3.8% (*w*/*v*) sodium citrate at a ratio of 1:9. The samples were centrifuged at 3000 rpm for 10 min, and the supernatant was absorbed to separate platelet-poor plasma (PPP). The chitosan-clay material was mixed with plasma at a ratio of 2 mg/100 μL, and the supernatant was extracted by centrifugation. Sample plasma (50 μL) was mixed with 50 μL of activated partial thromboplastin time (APTT) reagent and a steel ball, and incubated at 37 °C for 3 min. A CaCl_2_ (50 μL) reagent was added, followed by clotting time measurement using a semiautomatic coagulation analyzer (SteellexSC40, GTMsteel Science, Shenzhen, China). Steel balls were added to sample plasma (50 μL) and incubated at 37 °C for 3 min, 100 μL prothrombin time (PT) reagent was added, the solution was measured by a semiautomatic coagulation analyzer, and the time was recorded [47].

### 3.6. Whole Blood Clotting Tests

Several 5 mL centrifuge tubes of the same specification were taken, autoclaved at 121 °C for 30 min, and dried for later use. A 30 mg test sample was accurately weighed and placed in a centrifuge tube. Celox powder and a blank centrifuge tube without sample-treated groups were used as positive and negative controls, respectively. New Zealand rabbits were anesthetized with 3% (*w*/*v*) sodium pentobarbital intravenously at a dose of 1 mL/kg through the ear vein. The rabbits were supine and fixed. Femoral artery blood was collected, and 1 mL rabbit whole blood was immediately added to tube. The centrifuge tubes were placed in a water bath at 37 °C and observed every 15 s until blood flow had ceased completely, and the coagulation time was recorded [44]. The experiments were repeated three times, and the final clotting time was expressed as mean ± standard deviation (SD).

### 3.7. Erythrocyte Aggregation Test

A red blood cell solution of 5% specific volume was added with deionized water at a ratio of 1:4 (*w*/*v*) to lyse the red blood cell. The red blood cell was scanned successively at 200 nm~900 nm under an ultraviolet spectrophotometer, and the maximum wavelength was 540 nm. The red blood cells of 5% specific volume were divided into two parts, for UV spectrophotometry and scanning electron microscopy, respectively. Hemostatic material (100 mg) was placed in a 10 mL centrifuge tube, 5 mL of red blood cell suspension with 5% specific volume was added to each tube, and it was incubated at 37 °C for 10 min. The suspension soaked in 3 mL hemostatic material was added to deionized water at a ratio of 1:4 (*w*/*v*) to cleave red blood cells. The absorbance value was measured at 540 nm. Without sample as negative control. Each of the above samples was measured three times. The hemostatic material was directly immersed in 5% specific volume red blood cell suspension, and incubated at 37 °C for 1 h. The hemostatic material was washed with PBS three times to remove the red blood cells physically adsorbed on the surface. The material was fixed with 2.5% glutaraldehyde at 4 °C for 8 h. Then, gradient elution was performed with 20%, 50%, 80%, and 100% ethanol solutions. Then, the material was dried naturally at room temperature and observed under a scanning electron microscope [47].

### 3.8. Thromboelastograph Analysis

Anticoagulated whole blood was mixed with hemostatic material at a ratio of 10 mg/mL and incubated at 37 °C for 1 min. Then, 1 mL of the blood was sucked out and mixed with TEG reagent 1, and incubated for 3 min. In the next step, 340 uL of reagent-1-activated blood was mixed with reagent 2 (CaCl_2_) and tested using thromboelastometry (UD-T5000, China). The negative control group was anticoagulated whole blood with sodium citrate without sample [25,47].

### 3.9. Hemostatic Efficiency on Rat Tail Amputation

The hemostatic properties of the samples were tested and evaluated using the rat tail amputation model [48]. Twenty-four healthy male Sprague-Dawley rats (180~220 g, *n* = 6 per group) were anesthetized with 3% sodium pentobarbital (50 mg/kg) prior to surgery. Fifty percent of the length of the tail was cut with surgical scissors. After 15 s of free bleeding, the cut tail was placed in a centrifuge tube containing the sample to cover the wound with powder completely and gently pressed to stop the bleeding. The bleeding was observed every 15 s until the cut tail ceased bleeding, and hemostasis time and blood loss were recorded. Axenic gauze treatment was used as a blank control group, and Celox treatment was used as the positive control group. At the end of the experiment, rats were killed by euthanasia.

### 3.10. Hemostatic Efficiency on the Rat Arteriovenous Injury Model

The hemostatic performance was further evaluated using the rat femoral arteriovenous injury model [49,50]. Twenty-four healthy male Sprague-Dawley rats (180~220 g, *n* = 6 per group) were anesthetized with 3% sodium pentobarbital (50 mg/kg) before surgery and fixed supine on the experimental operating table. The surface of the left leg of rats was shaved using electric clippers, and the skin was disinfected with alcohol. An incision was made at the anterior groin of the left leg to expose the arteriovenous vessels, and the arteriovenous vessels were totally transected to create injury and hemorrhage. After 5 s of natural bleeding, the bleeding blood was absorbed with axenic gauze, and the wound was completely covered in powder and pressed with the same pressure (50 g weight). The bleeding was observed every 15 s until bleeding ceased, and hemostasis time and blood loss were recorded. Normal saline was used to clean the wound carefully. Finally, the histological samples were collected around the wound site with the femoral arteriovenous vessels, after euthanizing with an overdose of pentobarbital, and tissues were fixed in 4% phosphate buffered-paraformaldehyde. Paraffin sections were prepared, stained with hematoxylin-eosin (HE), and observed microscopically.

### 3.11. Hemolysis Test In Vitro

Rabbit blood was used to evaluate the influence of composite material on in vitro hemolysis. Fresh anticoagulant New Zealand rabbit blood (5 mL) was added to normal saline (5 mL) and diluted for later use. The sample groups included K, MMT, CS, CSK22, and CSMMT22. Chitosan was mixed with kaolin and montmorillonite in a 1:1 ratio without treatment, and coded as CS/K and CS/MMT, respectively. Diluted anticoagulant rabbit blood (200 μL) was added to 5 mL NaCl solution (0.9%) with each 50 mg sample, followed by incubation at 37 °C for 1 h. Then, the mixtures were centrifuged at 3000 rpm for 5 min. The absorbance was measured at 545 nm using a UV-Vis spectrophotometer (UV-2600, Shimadzu, Japan) [41]. Deionized water and normal saline were set as the positive and negative control, respectively. The hemolytic ratio was calculated by Equation (1):

Hemolytic ratio (%) = (Test sample − Negative control)/(Positive control − Negative control) × 100%(1)

### 3.12. Cytotoxicity Assay

The cytotoxicity of CSK and CSMMT was evaluated by MTT assay using the L929 mouse fibroblast cell line [44]. The samples were sterilized via Co-60 *γ*-irradiation at a dose of 25 kGy. Serum-free DMEM was used as the extraction medium, and the extraction ratio was 0.2 g/mL at 37 °C for 24 h. L929 cells at a density of 1 × 10^4^ per well were added to a 96-well plate and incubated for 24 h for cell attachment. The sample extract was supplemented with 10% FBS, and incubated for 24 and 48 h instead of medium. After the interaction, 50 µL MTT solutions (1 mg/mL) were used for another 2 h exposure. The extractant medium was drawn out, and 100 µL isopropanol was added to dissolve the formazan crystals. A microplate reader (SpectraMax 190, Molecular Devices, Sunnyvale, Silicon Valley, USA) was used to measure the absorbance of formazan solution at 570 and 650 nm (calibration wavelength). Cells in the fresh culture medium were used as the negative control, and cells in the culture medium that contained 0.2% (*w*/*v*) phenol were used as the positive control. Cell viability was represented with relative growth rate (RGR; %), calculated using Equation (2):

RGR (%) = (Test sample_570–650_)/(Negative control_570–650_) × 100% (2)

### 3.13. Acute Toxicity Test

Eighteen healthy male Kunming mice (18~22 g, *n* = 6 per group) were used for the acute toxicity test. Saline was used as the extraction medium, and the extraction ratio was 0.2 g/mL at 37 °C for 24 h. Animals in the experimental group were given 50 mL/kg of experimental sample extract by intraperitoneal injection, while the negative control group was given saline. The systemic toxicity of all mice from 1 to 7 days after administration was observed and recorded, and the mice were weighed daily from the beginning of the experiment to the end of the experiment [51].

### 3.14. Skin Irritation Test

Three healthy male New Zealand rabbits (2.0~2.5 kg, *n* = 3 per group) were used for the skin irritation test. Saline was used as the extraction medium, and the extraction ratio was 0.2 g/mL at 37 °C for 24 h. One day before the experiment, the hair on both sides of the back spine of New Zealand rabbits was removed with a depilator. Sterile gauze was cut into 4 layers of 2.5 cm × 2.5 cm gauze blocks. The experimental sample group was soaked with extract, and the negative and positive controls were soaked with saline and 20% sodium dodecyl sulfate, respectively. Medical sealing tape fixed the contact part for 24 h. At the end of the contact period, the patch was removed, and the skin conditions of each stimulated site were observed and recorded at 24 h, 48 h and 72 h.

### 3.15. Statistical Analysis

GraphPad Prism version 8.0 was used to perform statistical analysis. One-way analyses of variance were conducted followed by Student’s tests, where *p* < 0.05 indicated statistically significant data and *p* < 0.01 indicated extraordinary significant data.

## 4. Conclusions

In this study, the novel hemostatic agents CSK and CSMMT were successfully prepared and characterized by FTIR and XRD. After reacting with chitosan, the zeta potential of the composite was positively charged. Due to the synergistic hemostasis effect of chitosan and clay, CSK and CSMMT exhibited excellent in vitro hemostasis ability aided by APTT, PT, in vitro clotting time, RBCs aggregation, and TEG. The rat tail amputation model and arteriovenous hemorrhage experiments showed that, compared to Celox, the hemostasis time of CSK22 was reduced by 21.9% and 39.1%, respectively, and blood loss was reduced by 12.3% and 46.4%, respectively. The hemostasis time of CSMMT22 was reduced by 26.0% and 21.7%, respectively, and blood loss was reduced by 23.1% and 37.2%, respectively. Histological analysis showed that there was no inflammatory infiltration and pathological degeneration in the blood vessels of the rat arteriovenous injury model. In safety studies, CSK22 and CSMMT22 did not cause acute toxicity and skin irritation. More importantly, the composite material can ameliorate hemolysis and cytotoxicity caused by kaolin and montmorillonite. This preliminary study showed that CSK22 and CSSMMT22 had better hemostatic potential than the chitosan hemostatic agent Celox, a safe and effective hemostatic agent.

## Figures and Tables

**Table 1:** Elemental composition of K, K−DMSO, CSK42, CSK22, CSK12, MMT, CSCMMT42, CSMMT22, and CSMMT12 (wt %).

Element	K	K−DMSO	CSK42	CSK22	CSK12	MMT	CSMMT42	CSMMT22	CSMMT12
Si	25.47	22.86	25.37	25.37	25.42	34.44	37.05	36.46	36.09
Al	22.88	20.82	22.68	22.86	22.86	7.510	6.910	6.920	6.890
Ca	0.023	0.020	0.006	0.003	0.004	1.380	0.510	0.507	0.517
Na	0.205	0.175	0.016	0.017	0.014	1.590	0.460	0.471	0.463
S	0.022	3.950	0.111	0.101	0.102	0.006	0.010	0.014	0.006
Fe	0.312	0.261	0.328	0.319	0.298	0.646	0.687	0.667	0.575

**Table 2:** In vitro TEG results. Data represent the mean ± SD (*n* = 3). * *p* < 0.05; ** *p* < 0.01 compared to Control.

Samples	R (min)	K (min)	Angle (Deg)	MA (mm)
Control	2.40 ± 0.30	0.87 ± 0.12	78.03 ± 1.99	80.13 ± 1.81
CS	2.23 ± 0.35	0.80	79.87 ± 1.18	83.60 ± 4.87
K	0.37 ± 0.12 **	0.80	84.07 ± 0.98 *	83.73 ± 2.02
MMT	0.40 ± 0.10 **	0.80	84.90 ± 1.82 *	86.60 ± 7.61 *
CSK22	0.50 ± 0.17 **	0.80	84.03 ± 1.42 *	86.27 ± 5.80 *
CSMMT22	0.33 ± 0.06 **	0.80	85.53 ± 1.05 **	86.97 ± 6.47 *

## Data Availability

Not applicable.

## Sample Availability

Samples of hemostatic material are available from the authors.
